# The treatment role of *Cyperus rotundus* L. to triple-negative breast cancer cells

**DOI:** 10.1042/BSR20190502

**Published:** 2019-06-07

**Authors:** Fukai Wang, Xiang Song, Shuangshuang Ma, Chenyu Liu, Xiaohui SUN, Xinzhao Wang, Zhaoyun Liu, Dong Liang, Zhiyong Yu

**Affiliations:** 1 Department of Oncology, Shandong Cancer Hospital affiliated to Shandong University, Shandong Academy of Medical Sciences, Jinan, Shandong 250017, China; 2 Shandong University of Traditional Chinese Medicine, Jinan, Shandong 250014, China; 3 School of Medicine and Life Sciences, University of Jinan-Shandong Academy of Medical Sciences, Jinan, Shandong 250017, China; 4 Shandong Analysis and Test Center, Qilu University of Technology (Shandong Academy of Sciences), Jinan, Shandong 250014, China; 5 Department of Physiology, Georgetown University School of Medicine, Washington, DC 20057, U.S.A.; 6 Department of Breast Surgery, Affiliated Hospital of Shandong University of Traditional Chinese Medicine, Jinan, Shandong 250014, China

**Keywords:** Apoptosis, Autophagy, Cyperus rotundus L., Triple-negative breast cancer

## Abstract

*Cyperus rotundus* L. is widely used in Traditional Chinese Medicine and studies have reported its anticancer effect, but its chemical composition and therapy mechanism remains unknown. This research aims to analyze the chemical components of the ethanol extract of *Cyperus rotundus* L. (EECR), detect its treatment effects on human Triple-negative breast cancer (TNBC) cells, and elucidate possible therapy mechanisms. The chemical components of EECR were detected by the Waters UPLC combined with Bruker Q-TOF mass spectrometer (UPLC-Q-TOF-MS). The phytochemical compounds were identified by comparing the mass fragmentations of each metabolite with databases such as METLIN, HMDB, and NCBI. A total of 21 compounds were identified in EECR. MDA-MB-231 and MDA-MB-468 cells were treated with various concentrations of EECR. Cell proliferation was examined using Cell Counting Kit-8 (CCK-8) and colony formation assays. Cell apoptosis and cell cycle were detected by flow cytometry. Apoptosis- and autophagy-related protein expression was detected by Western blot. EECR inhibits the proliferation of TNBC cells (MDA-MB-231 and MDA-MB-468) in a dose-dependent manner, which may be related to the arrest of cell cycle in G_0_/G_1_ phase. It induces apoptosis by promoting the expression of BAX and inhibiting the expression of BCL-2. In addition, autophagy inhibitor 3-Methyladenine (3-MA) inhibited TNBC cells pro-survival autophagy and increased the sensitivity of EECR. The present results demonstrated that EECR has potential effects on inhibits the proliferation and induction apoptosis in TNBC.

## Introduction

There will be approximately 2.1 million newly diagnosed female breast cancer cases in 2018, accounting for 24.2% of female cancer cases [1]. Among women, breast cancer is the most commonly diagnosed cancer and the leading cause of cancer deaths, accounting for 15% of female cancer deaths. Triple-negative breast cancer (TNBC) is defined as the negative immunohistochemical test results of estrogen receptor (ER), progesterone receptor (PR), and human epidermal growth factor receptor-2 (Her-2) [2,3]. Due to the specific molecular expression characteristics of TNBC, these patients are resistant to existing endocrine therapy and Her-2-targeted therapy [4], therefore endocrine therapy and Her-2 targeted therapy are not suitable for TNBC patients [5]. So far, treatment options for TNBC are limited, seldom benefit from chemotherapy and easy resistance. TNBC has a poor prognosis with higher rates of recurrence and metastasis compared with other types of breast cancer. As the age of TNBC patients is trending younger [6], the discovery of new and effective drugs is needed for the treatment of TNBC.

Traditional Chinese Medicine mainly coming from plants has a history of more than 2000 years. We intend to screen an effective drug from Traditional Chinese Medicine for the treatment of TNBC, providing an alternative clinical option. The dry rhizomes of *Cyperus rotundus* L. named Xiangfu have been applied for more than 1700 years in China, mainly being applied for the treatment of gynecological diseases. Current pharmacological studies have shown that it has significant neuroprotective, antioxidant, anti-DNA damage, antibacterial, and anti-diabetic effects [7–13]. According to ancient literature, Xiangfu could be ground into powder, mixed with ginger juice and wine for external application to treat breast cancer. Park et al. [14] reported that ethanol extract from the dry rhizomes of *C. rotundus* (EECR) can induce apoptosis of MDA-MB-231 cells, but the potential molecular mechanism and chemical components of EECR still remain unknown.

Owing to Xiangfu’s complex chemical composition, its bioactives may play a role at various types of sites such as cell cycle arrest, autophagy, and apoptosis in the cancer treatment [15–17]. Some research supported that the induction of cell cycle arrest might be a valid way of controlling cancer cell proliferation [18–20].

Autophagy is an intracellular process that enables cells to recover components, like damaged proteins and organelles, by a controlled pathway [21]. Morphologically, the characteristic manifestation of autophagy is the early formation of isolation membrane, which forms autophagosome and this process is mediated by LC3. Lysosome combines with the newly formed autophagosome and the content enclosed is digested [22,23]. Beclin-1 can induce autophagy, which is an important protein in the initiation of autophagosome formation [24,25], and LC3 is an essential protein in the final stage [26]. Autophagy played a key role in pro-survival and pro-apoptosis, while apoptosis ultimately leads to cell death [27]. Both Bax and Bcl-2 belong to the Bcl-2 family, the former has the role of pro-apoptotic, whereas the latter plays anti-apoptotic roles [28–30]. However, the association between apoptosis and autophagy is complex.

In the present study, we found that the EECR induces apoptosis and the autophagic activity changed in TNBC cells. We will expound the relationship between apoptosis and autophagy. Meanwhile, we analyze the chemical components of the EECR, lay the foundation for extracted effective constituents to treat TNBC.

## Materials and methods

### Pharmacological reagents

The dry rhizomes of *C. rotundus* were purchased from Anhui Xiehecheng Co., Ltd. (Bozhou, China) and 3-Methyladenine (3-MA) was purchased from Selleckchem (Houston, U.S.A.). 3-MA was dissolved in dimethyl sulfoxide (DMSO; Thermo Fisher Scientific, Massachusetts, U.S.A.). In all cases of cell treatment, the final DMSO concentration never exceeded 0.3% in the culture medium. Stock solutions of all drugs were stored at −80°C.

### Plant material and extract preparation

The dry rhizomes of *C. rotundus* were cut into small pieces, transferred to a round-bottomed flask at a ratio of 1:10 (drug:95% ethanol, w/v), and immersed in the dark for 12 h at room temperature. The EECR was prepared by refluxing and extracting in a water bath at 80°C for 2 h, the supernatant was obtained by the process of vacuum suction filtration. Repeat the above test for another two times. The supernatants were mixed and the EECR was achieved by reduced pressure distillation at 40°C. The EECRs were lyophilized and stored at −80°C for the following experiment.

### Mass spectrometry analysis of EECR

The EECR was dissolved with methanol at a concentration of 80 mg/ml and analyzed by the Waters UPLC (Acquity UPLC class, U.S.A.) combined with Bruker Ultra-High Resolution Quadrupole-Time-Of-Flight mass spectrometer equipped with ESI interface (Bruker Impact II™, Germany). The LC analyses were performed on a C18 column (XDB-C18 4.6 mm × 100 mm 1.8 μm) at the temperature of 35°C. The mobile phase A consisted of water and 0.1% formic acid, while the mobile phase B included acetonitrile and 0.1% formic acid. A gradient elution was optimized and the proportion of phase B was confirmed as follows: 0–1 min, 5%; 1–60 min, 5–40%; 60–75 min, 40–57%; 75–87 min, 57–95%; 87–87.5 min, 5%; 87.5–95 min, 5%. The flow rate was 0.5 ml/min. The injection volume was 5 μl. Positive ion models were used in the detection. Capillary voltage was 4500 V; nebulizer pressure was 2.0 Bar; the flow rate of day gas was 8.0 l/min and the drying gas temperature was 200°C. The range of mass scan was from m/z 50 to 1000.

### Cell culture and treatment

TNBC cell lines (MDA-MB-468 and MDA-MB-231) were purchased from the Cell Bank of Shanghai Institute of Cell Biology (Chinese Academy of Sciences). Cells were cultured in Dulbecco’s modified Eagle’s medium (DMEM) (high glucose, KeyGen Biotech, Jiangsu, China) supplemented with 10% fetal bovine serum (Gibco, New York, U.S.A.) and 100 units/ml antibiotics (penicillin and streptomycin, KeyGen Biotech, Jiangsu, China) in a humidified atmosphere of 5% CO_2_ at 37°C.

### Cell counting kit-8 assay

The Cell Counting Kit-8 assay (CCK-8) (MedChemExpress, New Jersey, U.S.A.) was used to determine cell viability following treatment with a corresponding concentration of EECR. MDA-MB-231 and MDA-MB-468 cells were seeded at a density of 1 × 10^4^ cells/well in 96-well plates and treated with various concentrations of EECR for 24 h. CCK-8 was added to each well according to the instructions, followed by incubation at 37°C in 5% CO_2_ for 1 h. Then, the absorbance was examined using a microplate reader (SpectraMax i3; Molecular Devices, Sunnyville, U.S.A.) at 450 nm, and displayed the optical density (OD). Cell viability was assessed using CCK-8 assay according to the manufacturer’s instructions.

### Colony formation assay

MDA-MB-231 and MDA-MB-468 cells were plated into a six-well plate at a concentration of 800 cells/well and treated with a corresponding concentration of EECR for 24 h and then incubated at 37°C for 14 days. Then the cells were fixed with the fixation solution (methanol:acetic acid = 1:3) and stained with Crystal Violet. The number of colonies (>50 cells) was counted.

### Analysis of cell cycle and apoptosis by flow cytometry

Cells treated with EECR for 24 h were collected using EDTA-free trypsin, centrifuged (1000 rpm, 3 min) and washed twice with cold PBS for the detection of apoptosis and cycle. The cells for detecting apoptosis were subjected to the following procedures: the cells were re-suspended with 1× binding buffer at a density of 1 × 10^6^ cells/ml. One hundred microliters of above-mentioned cell suspension was treated with 5 μl of Annexin V-FITC and 5 μl of Propidium Iodide (PI) and incubated at 4°C for 30 min in the dark. The cells for the cycle detection were operated as follows: the cells were re-suspended with pre-cooled 70% ethanol, allowed to stand at 4°C for 72 h, and washed twice with cold PBS. Three hundred microliters of PI solution was added to the treated cells and incubated at 4°C for 30 min in the dark. Flow cytometric analysis was performed to analyze cell-cycle distribution.

### Western blot

After treating the cells with different concentrations of EECR for 24 h, the original medium was discarded. The adherent cells were washed with cold PBS on the ice and lysed in cell lysis buffer (Beyotime, Beijing, China) containing 0.5 mM phenylmethanesulfonyl fluoride (PMSF, Beyotime, Beijing, China) for 20 min. The suspension was centrifuged at 12000 rpm for 20 min at 4°C, and the pellet was removed. The BCA Protein Assay Kit was used to determine the protein concentration of the supernatant. Then the supernatant was mixed with protein loading buffer and boiled at 100°C for 5 min. Twenty microgram proteins were fractioned by sodium dodecyl sulfate/polyacrylamide gel (SDS/PAGE) for acquiring proteins with specific molecular weight and then electro-transferred to a polyvinylidene difluoride (PVDF) membrane, blocked in 5% evaporated skim milk for 1 h and incubated with the following primary antibodies: β-Actin (4970T), BAX (2772T), Bcl-2 (4223T), Beclin-1 (3495T), LC3B (2775S) (all 1:1000; Cell Signaling Technology, Massachusetts, U.S.A.) overnight at 4°C. Samples were washed three times for 6 min each and were incubated with a horseradish peroxidase–conjugated second anti-rabbit antibody for 1 h at room temperature. Finally, protein expression was detected using an enhanced chemiluminescence system (Bio-Rad, U.S.A.).

### Statistical analysis

All data were analyzed by SPSS 17.0 software, ImageJ software 1.42, and GraphPad Prism 7.0. Student’s *t* test and one-way ANOVA were used to analyze statistical significance. *P*<0.05 was considered to indicate statistical significance. Three independent experiments were performed under the same conditions.

## Results

### Phytochemical analysis of EECR

**Figure 1:**
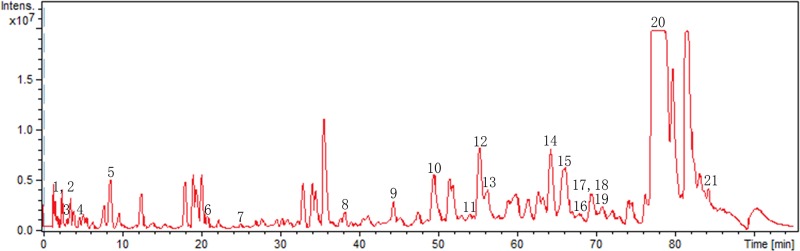
The base peak chromatogram (BPC) of the EECR

The EECR was analyzed by the UPLC combined with Bruker Q-TOF mass spectrometer (UPLC-Q-TOF-MS) to earn the metabolites profile of the EECR and the Base Peak Chromatography was shown in Figure 1. The phytochemical compounds were identified by comparing the mass fragmentations of each metabolite with databases such as METLIN (https://metlin.scripps.edu), HMDB (http://www.hmdb.ca/), and NCBI (https://www.ncbi.nlm.nih.gov/). A total of 21 compounds were identified. The detailed information containing mass fragmentations is listed in Table 1.

**Table 1:** Phytochemical compounds information identified from the EECR

ID	Real time	Intension	S/N	m/z (calculate)	MS[M+H]^+^/ [M+Na]^+^	MS/MS	Molecular formula	Tentative assignment
1	2.6	366163	282.8	109.0522	110.0606	94.0291 (30.4); 80.0492 (14.9)	C_6_H_7_NO	3-Hydroxy-2-Methylpyridine
2	2.6	1078647	828.3	123.0315	124.0392	80.0498 (50.9); 96.0445 (24.1)	C_6_H_5_NO_2_	4-Carboxypyridine
3	2.8	3779137	2462.2	267.0962	268.1041	136.0617 (100); 119.0353 (5.9)	C_10_H_13_N_5_O_4_	Vidarabine
4	4.2	281429	255.7	143.0399	144.0476	113.0294 (100); 112.0214 (69.5); 126.0376 (15.4)	C_6_H_9_NOS	4-Methyl-5-Thiazoleethanol
5	8.8	144537	258.7	117.0578	118.065	91.0539 (100); 117.0574 (58); 89.0387 (53.3)	C_8_H_7_N	Indole
6	20.4	5488495	2698.6	233.1774	234.185	146.0962 (100); 147.0995 (9.8); 131.0729 (6.4)	C_15_H_23_NO	5-Hydroxyiminoisocaryophyllene
7	25.3	494415	195.5	134.109	135.1167	91.0543 (100); 105.0698 (41.4)	C_10_H_14_	1,3,8-Menthatriene
8	38.4	1816029	1146.7	286.0472	287.0552	153.0184 (16.2); 135.0443 (4.9)	C_15_H_10_O_6_	3′,4′,5,7-Tetrahydroxyflavone
9	43.1	551334	336.3	108.057	109.0648	94.0415 (100); 107.0491 (16.3); 91.0549 (14.2)	C_7_H_8_O	4-Methylphenol
10	49.6	5048862	364	234.1614	235.1692	105.0699 (100); 119.0854 (67.8); 133.1011 (52.4)	C_15_H_22_O_2_	Confertifoline
11	54.7	1439981	221.3	250.1563	251.164	133.1009 (100); 105.0698 (71.5); 119.0854 (63.3); 149.0595 (61); 145.1008 (51.3)	C_15_H_22_O_3_	3β-Hydroxycinnamolide
12	55.5	1663269	406	234.1614	257.1509	105.07 (100); 119.0862 (86.8); 107.0855 (84.4); 133.101 (81); 145.1015 (80.4); 131.0855 (68.3); 91.0542 (59)	C_15_H_22_O_2_	Macrophyllic acid A
13	56.3	3321996	239.5	232.1463	233.1533	105.0699 (100); 119.0855 (75); 91.0543 (63.8); 107.0855 (59.5); 161.0963 (57); 133.1009 (51.5)	C_15_H_20_O_2_	Isoalantolactone
14	64.4	7846727	1276.6	236.1771	237.185	119.0854 (100); 105.0699 (83.4); 161.1323 (74.4); 107.0855 (65.6); 135.1168 (63.9)	C_15_H_24_O_2_	Petasitolone
15	66.2	5495935	605.9	216.1509	217.1586	119.0854 (100); 105.0698 (47.7); 133.1011 (29.8); 91.0543 (28.8)	C_15_H_20_O	Aromatic turmerone
16	67.9	1582196	347.1	248.1407	249.1488	133.1009 (100); 119.0854 (91.9); 203.1428 (79.2); 105.0699 (79.1); 175.1477 (68.8)	C_15_H_20_O_3_	Illudin M
17	69.6	2604122	286.6	218.1665	219.174	105.0698 (100); 119.0854 (71.5); 91.0543 (68); 145.1008 (57.1); 93.07 (52.9); 121.1008 (51.1)	C_15_H_22_O	Isohumbertiol
18	69.6	1315343	403.3	236.1771	259.1666	145.1013 (100); 119.0854 (63); 105.0702 (56.4)	C_15_H_24_O_2_	Capsidiol
19	70.9	1306179	249.4	200.156	201.1635	105.07 (100); 131.0852 (92.9); 145.1007 (70.2); 129.0694 (64.5); 91.0543 (61); 119.0853 (51.3)	C_15_H_20_	α-Calacorene
20	78.6	3237428	1804.2	134.0726	135.0802	91.0544 (100); 115.054 (76.9)	C_9_H_10_O	3-Phenylpropanal
21	84.3	3119893	785.7	202.1716	203.1792	105.0699 (100); 119.0856 (64.9); 91.0543 (58.8)	C_15_H_22_	α-Curcumene

Each component was identified by comparing the MS/MS with databases, error ≤ 5 ppm.

### EECR suppressed proliferation of TNBC cells

**Figure 2:**
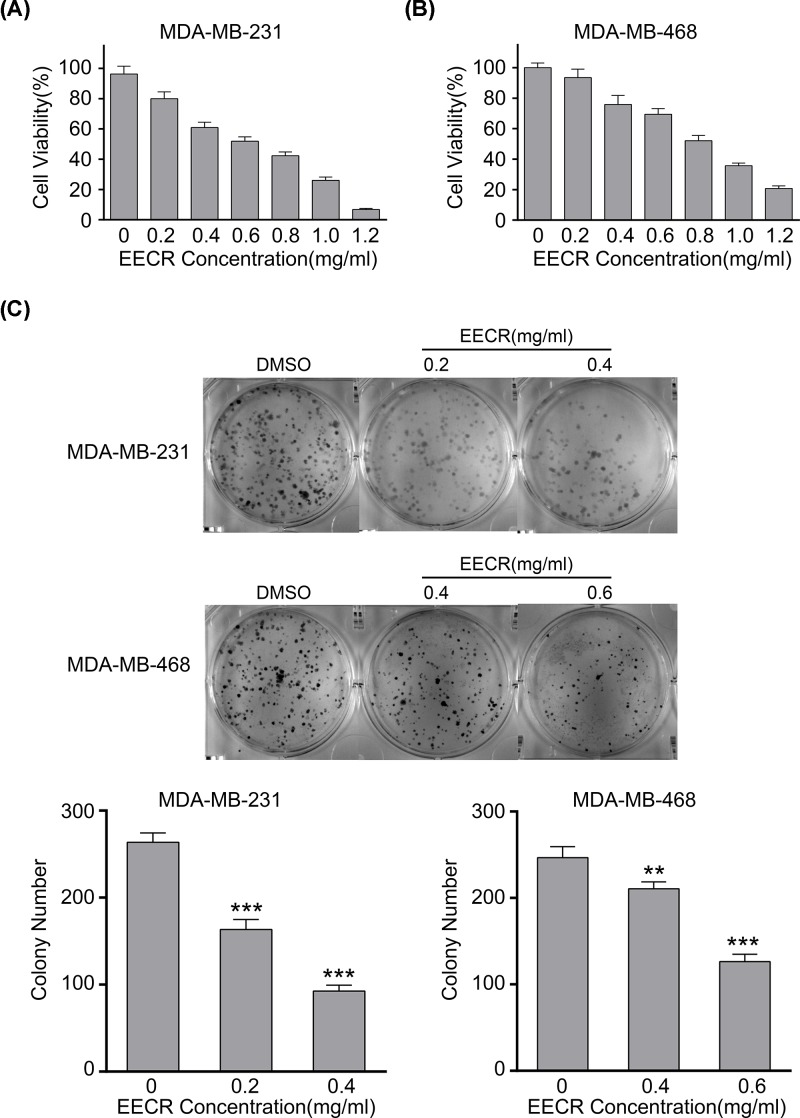
Inhibitory effect of EECR on the proliferation of TNBC cells (**A**) MDA-MB-231 and MDA-MB-468 cells were treated with 0, 0.2, 0.4, 0.6, 0.8, 1.0, and 1.2 mg/ml EECR for 24 h. Cell viability was determined by the CCK-8 assay. (**B**) MDA-MB-231 and MDA-MB-468 cells were seeded on six-well plates and treated with 0, 0.2, 0.4 and 0, 0.4, 0.6 mg/ml EECR for 24 h, respectively. (**C**) Colony formation was assessed by Crystal Violet staining after 14 days. The results were mean ± SD and three independent experiments were performed under the same conditions. *P*<0.05 was considered statistically significant compared with the control group. **P<0.05, ***P<0.001 compared with the control group.

To visualize the cytotoxicity of EECR to TNBC cells, MDA-MB-231 and MDA-MB-468 cells were treated with 0, 0.2, 0.4, 0.6, 0.8, 1.0, and 1.2 mg/ml EECR for 24 h. Exposed to EECR, cell viability was reduced in a concentration-dependent manner (Figure 2A,B). We selected three concentrations to treat MDA-MB-231 (0, 0.2, and 0.4 mg/ml) and MDA-MB-468 (0, 0.4, and 0.6 mg/ml) cells. The colony formation assay showed that the number of treatment group colonies was significantly lower than that of the control group (DMSO) (Figure 2C). The results of the CCK-8 assay demonstrated a similar trend. EECR inhibited the proliferation of TNBC cells in a dose-dependent manner.

### EECR induces apoptosis and G_0_/G_1_ arrest in human TNBC cells

**Figure 3:**
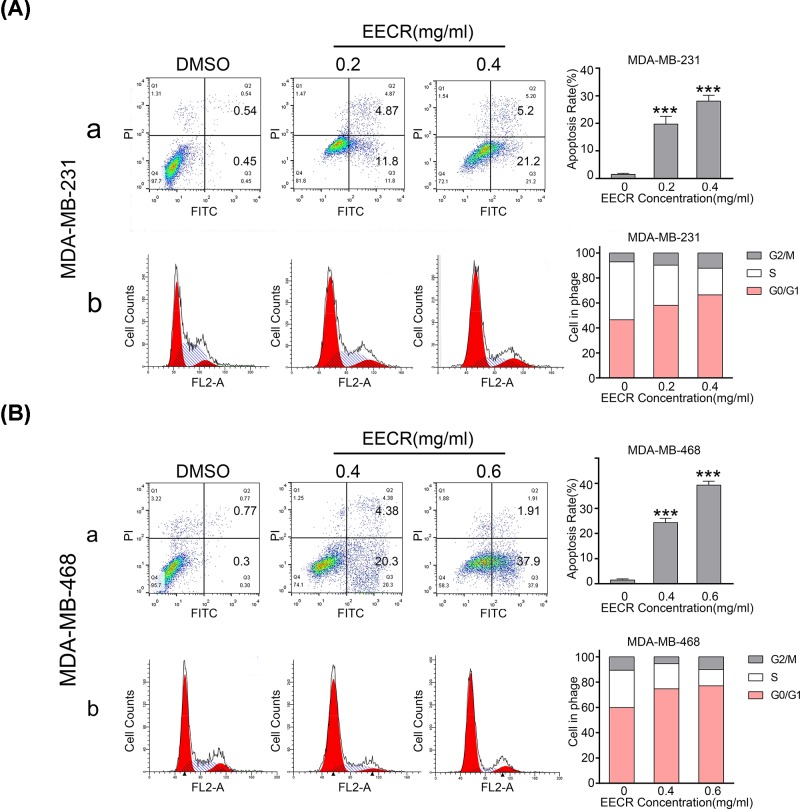
EECR induces TNBC cells’ apoptosis and increases the proportion of the G_0_/G_1_ phase in a concentration-dependent manner MDA-MB-231 and MDA-MB-468 cells were treated with corresponding concentrations of EECR for 24 h. (**A-a,B-a**) The Annexin V-FITC/PI assay was performed to determine the apoptosis proportion of different groups, and the results were analyzed by flow cytometry. (**A-b,B-b**) The DNA content of cells treated with different concentrations of EECR was determined by PI staining, and the results were analyzed by flow cytometry. The results were mean ± SD, three independent experiments were performed under the same conditions. *P*<0.05 was considered statistically significant compared with the control group. ***P<0.001 compared with the control group.

The effect of EECR on the cell cycle and apoptosis was shown in Figure 3. The EECR exposed group exhibits a higher apoptosis rate than the control group both in MDA-MB-231 (0, 0.2, and 0.4 mg/ml) and MDA-MB-468 (0, 0.4, and 0.6 mg/ml) cells (Figure 3A-a,B-a). As shown in Figure 3A-b,B-b, both TNBC cell lines showed an increased proportion of cells in the G_0_/G_1_ phase after EECR treatment compared with the control group. These results indicated that EECR induced apoptosis in MDA-MB-231 and MDA-MB-468 cells and prevents the cell cycle from proceeding to the M phase by halting them at the G_0_/G_1_ phase.

### Increased autophagy after EECR stimulation

**Figure 4:**
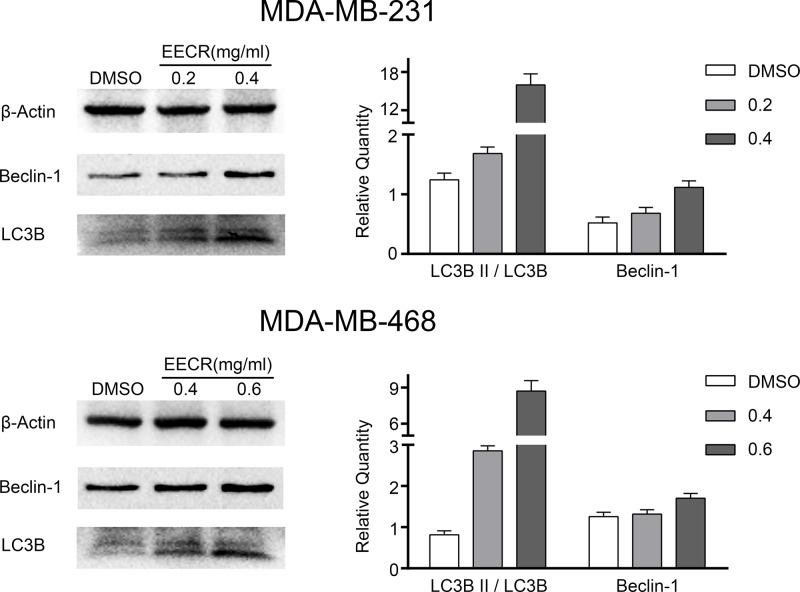
The expression level of autophagy-related proteins (Beclin-1, LC3B) in experimental and control groups assayed using Western blot Proteins were detected using the corresponding antibodies and normalized to β-Actin. Beclin-1 was high expression compared with control group. The conversion of LC3B I into LC3B II was increased. The results were mean ± SD, three independent experiments were performed under the same conditions.

The conversion of LC3B I into LC3B II, as a classic indicator of autophagy detection, was detected using Western blot. Proteins extracted from MDA-MB-231 (0, 0.2, and 0.4 mg/ml) and MDA-MB-468 (0, 0.4, and 0.6 mg/ml) cells were used as samples. Autophagy was up-regulated in the EECR treatment group, which was manifested by the high expression of Beclin-1 and high-ratio of LC3B II/LC3B I (Figure 4).

### Autophagy inhibitor enhances the cytotoxicity of EECR

**Figure 5:**
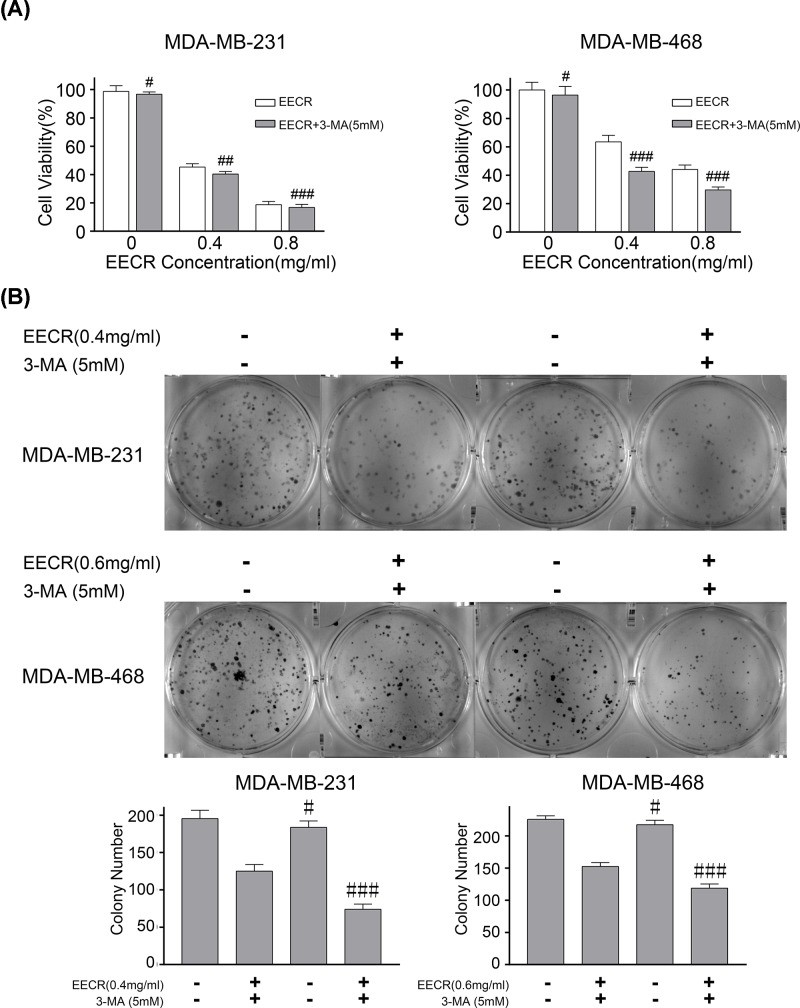
Effect of EECR on MDA-MB-231 and MDA-MB-468 cells under the existence of 3-MA After adding autophagy inhibitor 3-MA for half an hour, MDA-MB-231 and MDA-MB-468 cells were treated with EECR at 0.4 and 0.6 mg/ml for 24 h, respectively. (**A**) Comparing the group of EECR and 3-MA+EECR, cell activity of the latter group was significantly reduced after the addition of autophagy inhibitor. (**B**) Comparing with the EECR group, the cell proliferation of 3-MA+EECR group was significantly reduced. The results were mean ± SD and three independent experiments were performed under the same conditions. *P*<0.05 was considered statistically significant compared with the no autophagy inhibitor group; ^#^*P*>0.05, ^##^*P*>0.05, ^###^*P*<0.001 compared with the group of without 3-MA.

**Figure 6:**
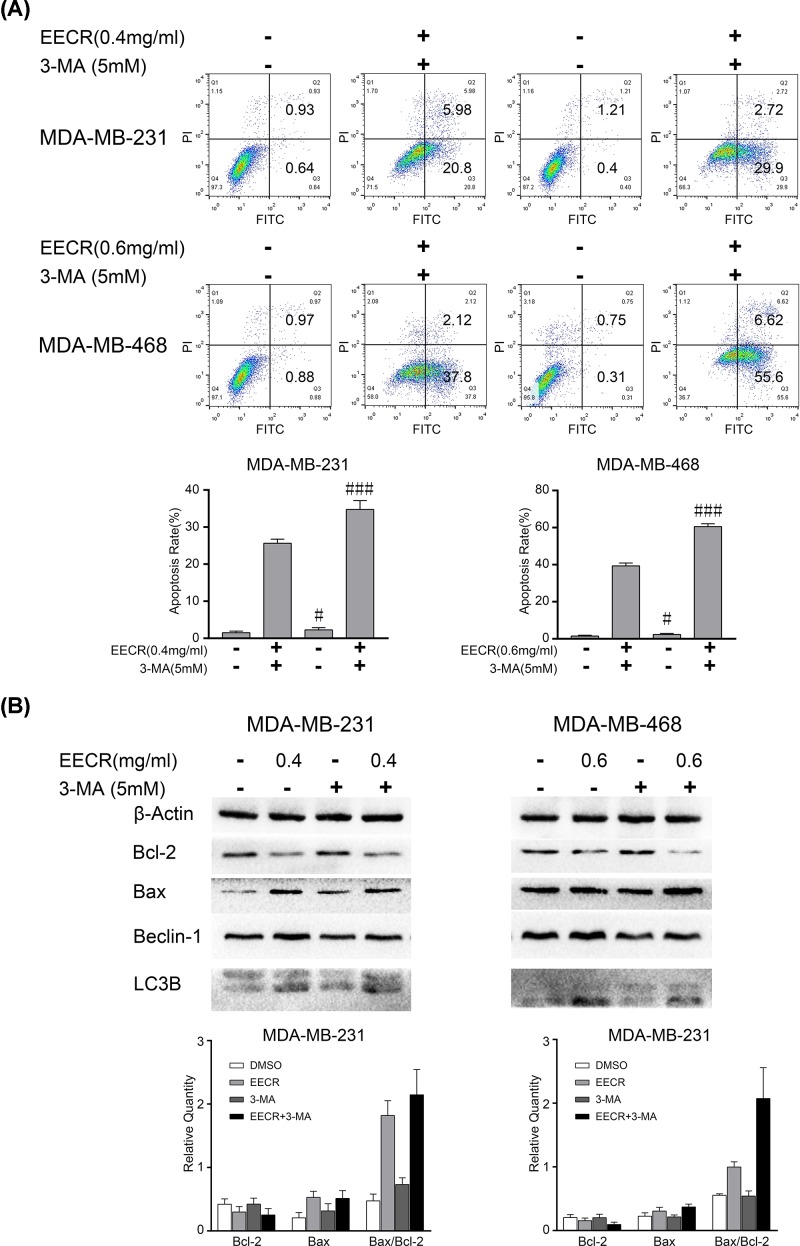
Effect of EECR with or without 3-MA on TNBC cells After adding 3-MA for half an hour, MDA-MB-231 and MDA-MB-468 cells were treated with EECR at 0.4 and 0.6 mg/ml for 24 h, respectively. (**A**) The flow cytometry results showed that the apoptotic rate was significantly increased after the addition of the autophagy inhibitor. (**B**) The results of Western blotting were consistent with those of flow cytometry. The results were mean ± SD and three independent experiments were performed under the same conditions. *P*<0.05 was considered statistically significant compared with the no autophagy inhibitor group, ^#^*P*>0.05, ^###^*P*<0.001 compared with the group of without 3-MA.

Comparing the group of EECR (0, 0.4, and 0.8 mg/ml) and EECR+3-MA, cell activity of the latter was significantly reduced (*P*<0.001) (Figure 5A,B). In addition, the flow cytometry results showed that the apoptotic rate was significantly increased in the EECR+3-MA group (MDA-MB-231 0.4 mg/ml and MDA-MB-468 0.6 mg/ml respectively), which was consistent with the colony formation (Figure 6A). As described in Figure 6B, the results of Western blot were similar to those of flow cytometry. Taken together, the results showed that pro-survival autophagy occurs in cells after addition of EECR, and autophagy inhibitor enhanced the cytotoxicity of EECR.

## Discussion

Unable to benefit from endocrine therapy and molecular targeted therapy, patients with TNBC have a poor prognosis. As the new cancer treatments continue to advance, drug resistance of tumor develops along. Traditional Chinese Medicine, which has a long history, has the advantage of sustainability and environmental protection. It is imperative to find active drugs from traditional Chinese medicines as an alternative treatment for patients.

According to the ancient Traditional Chinese Medicine literatures’ record, *Cyperus rotundus* L. is used to treat breast cancer. Various studies have identified that *C. rotundus* extraction has cytotoxic effects on different cancer cells. It has been reported to have cytotoxic effects on MCF-7 (breast cancer), HeLa (cervical cancer), HepG2 (liver cancer), PC-3 (prostate cancer), HT-29 (colorectal cancer) [31]. It can inhibit xanthine oxidase (XO), prevent DNA damage caused by lipid peroxidation and induce apoptosis of leukemia K562 cells and L1210 cells [32–34]. 11,12-dihydroxyeudesm-4-en-3-one isolated from the ethyl acetate extract of *C. rotundus* has significant cytotoxicity on ovarian cancer cell (A2780) [35]. 6-acetoxy cyperene isolated from *C. rotundus* can induce caspase-dependent apoptosis in ovarian cancer cells [36].

UPL-QTOF-MS is a high resolution and sensitive technology [37]. Recently, it has been widely applied in complicated component analysis [38]. We use UPLC-Q-TOF-MS to analyze the composition of EECR; a total of 21 compounds were identified by comparing the mass fragmentations of each metabolite with databases. Recent researches show that Indole, Isoalantolactone, and Aromatic-turmerone in EECR have anti-tumor activities [39–41]. Despite the fact that deep study is needed, these researches still provide support for our research.

For now chemical medication can treat cancer by inhibiting the proliferation and induce apoptosis of cells. Autophagy plays either pro-survival or pro-death roles in the cell’s life [42], which has different directive significance for the treatment of TNBC. In the current study, we explored the possible mechanism of EECR on the TNBC cells in proliferation, apoptosis, and autophagy, respectively.

Cell cycle out-of-balance is an important mechanism leading to the proliferation of cancer cells [43–45]. The common mechanism of anticancer drugs is that they can induce cell cycle arrest at specific stages and further induce apoptosis [43]. In the research, we found EECR can inhibit proliferation of TNBC cells, and the mechanism of action is relevant with cell cycle arrest in G_0_/G_1_ phase in a dose-dependent manner.

Apoptosis was the major reason of cell death induced by antitumor drugs. Bax and Bcl-2 are located on the outer membrane of mitochondria and belong to the pro-apoptotic Bcl-2 family and anti-apoptotic Bcl-2 family separately. An unbalanced Bax/Bcl-2 ratio can effectively promote the release of apoptotic effectors from mitochondria and drive apoptosis [46]. It is an important signal to evaluate cell apoptosis. In our study, we found that EECR induces TNBC cells’ apoptosis by flow cytometry in a concentration-dependent manner and the ratio of the Bax/Bcl-2 is increased. It suggested that EECR may induce apoptosis through mitochondrial-associated apoptotic pathway in TNBC cells.

Beclin1 plays a critical role in the autophagy regulation [47]. The ratio of the LC3-II/LC3-I as the common index is usually used to detect the degree of autophagy [48]. Thus, we detected the levels of Beclin1 and LC3 by Western blot after adding the EECR and found autophagy enhanced. Autophagy plays a role of pro-survival or pro-apoptosis in cells. To investigate the role of autophagy in cells, autophagy inhibitor 3-MA was added with or without EECR. We find apoptosis increased in the EECR+3-MA group compared with EECR group. This indicates that the autophagy plays a pro-survival role in TNBC cells, and autophagy inhibitor 3-MA could facilitate the EECR sensitivity by suppressing the pro-survival autophagy. It is consistent with our previous research, autophagy inhibitor facilitates gefitinib sensitivity [49].

In summary, the chemical composition of the EECR was analyzed by UPL-QTOF-MS. EECR inhibits the proliferation of TNBC cells (MDA-MB-231 and MDA-MB-468), which may be related to the arrest of cell cycle in G_0_/G_1_ phase. It induces apoptosis by promoting the expression of BAX and inhibiting the expression of BCL-2. Autophagy inhibitor 3-MA inhibited TNBC cells pro-survival autophagy and increased the sensitivity of EECR. These conclusions provide theoretical support for EECR in the treatment of TNBC. EECR contains abundant chemicals and we will further explore more effective anti-tumor active components, as well as more evidence from animal experiments.

## Availability of data and materials

All data generated or analyzed during the present study are included in this published article.

## Abbreviations

CCK-8: cell counting kit-8
DMSO: dimethyl sulfoxide
EECR: ethanol extract of *Cyperus rotundus* L.
Her-2: human epidermal growth factor receptor-2
PI: propidium iodide
TNBC: triple-negative breast cancer
UPLC-Q-TOF-MS: UPLC combined with Bruker Q-TOF mass spectrometer
3-MA: 3-methyladenine
